# Micro-Chamber/Thermal Extractor (µ-CTE) as a new sampling system for VOCs emitted by feces

**DOI:** 10.1038/s41598-021-98279-z

**Published:** 2021-09-21

**Authors:** Ileana Andreea Ratiu, Radik Mametov, Tomasz Ligor, Bogusław Buszewski

**Affiliations:** 1 Interdisciplinary Centre of Modern Technologies – BioSep, Nicolaus Copernicus University, Wileńska 4, 87-100, Toruń, Poland; 2 Department of Environmental Chemistry and Bioanalytics, Faculty of Chemistry, Nicolaus Copernicus University, Gagarina 7, 87-100 Toruń, Poland; 3 “Raluca Ripan” Institute for Research in Chemistry, Babes-Bolyai University, 30 Fantanele, 400239 Cluj Napoca, Romania

**Keywords:** Chemical tools, Gas chromatography, Statistical methods

## Abstract

VOCs (volatile organic compounds) are increasingly wished to be used in diagnosis of diseases. They present strategic advantages, when compared to classical methods used, such as simplicity and current availability of performant non-invasive sample collection methods/systems. However, standardized sampling methods are required in order to achieve reproducible results. In the current study we developed a method to be used for feces sampling using a Micro-Chamber/Thermal Extractor (µ-CTE). Design Expert software (with Box–Behnken design) was used to predict the solutions. Therefore, by using the simulation experimental plan that was further experimentally verified, extraction time of 19.6 min, at extraction temperature of 30.6 °C by using a flow rate of 48.7 mL/min provided the higher response. The developed method was validated by using correlation tests and Network analysis, which both proved the validity of the developed model.

## Introduction

VOCs emitted from feces are increasingly considered as potential useful markers in diagnostic of different diseases; however, standardization of sampling procedures is still in an incipient phase. Efforts for development of standardized sampling methods were made in different fields of metabolomics, and mostly for the case of breath samples^1–5^. Moreover, for breath analysis various sensors able to perform the analyses in real time, without any pre-concentration or storage, were created in the attempt to diagnose respiratory diseases^6–8^ and even a standardized breath sampler was developed^1^. Except breath samples, other matrices, such as urine^9–11^, sweat^12^, human tissues^13^, saliva^14^, breast milk^15^, exudates^16^, or bacteria^17–21^ were investigated in research articles and discussed in reviews^19,22–24^ in order to discover valuable biomarkers of certain diseases. In case of feces samples, literature in the field is related mostly to identification of those VOCs markers of colorectal-cancer^25–28^, but nevertheless considerably less progress has been made with feces compared with investigating other biological matrices, especially breath and urine. Optimization of sampling parameters for human feces’ samples was realized in terms of investigating collection procedure, transport, and storage^29–31^. Extraction of VOCs from fecal samples was also optimized, in terms of time (10 vs 20 min), storage at room temperature or freezing temperature, and amount of sample^32^. In terms of analytical response, the number of peaks obtained after performing GC–MS analyses was considered^32^. Nevertheless, to the best of our knowledge, no optimization of sampling parameters by using the Micro-Chamber/Thermal Extractor was realized before. Emitted components can be collected in a wide range of sorbent tubes and analyzed by standard analytical methods; usually a GC–MS or a GC–FID, equipped with a thermal-desorption (TD) unit, is being utilized. The µ-CTE was designed for a wide range of applications, including screening emissions from construction materials^33^, profiling aroma and fragrance of various foods and consumer products^34^. Nevertheless, µ-CTE can be successfully used for other applications, including sampling of VOCs emitted by various biological samples, as it was proved by the present study. In contrast to other studies, we used a complex optimization method, which has been able to provide the best possible solutions, predicted by software as a result of experimental measurements and total interactions between three selected factors. The selected factors were as follows: extraction temperature, extraction time and gas flow, while Design Expert software (with Box–Behnken design) was used to predict the solutions (responses).

The Box–Behnken design is an efficient and time saving optimization method, which can provide a representation of polynomial response function that cannot be described by linear functions. It can be applied for optimization parameters in many fields, and it was previously used in investigation of extraction parameters in accelerated solvent extraction^35^, pressurized liquid extraction^36^, effect of process parameters on the microparticle production^37^, evaluation of shale gas recovery enhancement^38^, etc.

In contrast with another previous study that realized kindred experiments^32^, in addition to number of peaks we investigated as desired responses total peaks’ area and peaks intensities. Consequently, three models were created, and then three final solutions were obtained. Our study included two steps: (1) optimization of sampling parameters (the main aim of the study) and (2) investigation of feces profiles coming from human volunteers of different ethnicities, genders, ages, with and without self-declared chronical diseases and having different culinary habits. To the best of our knowledge, the optimization of sampling parameters for µ-CTE was not investigated before for feces samples, or for other biological samples; this is why the present work represents an original approach.

## Materials and methods

### Materials and chemicals utilized

Analytical standards 4-bromofluorobenzene, alkanes (C8 to C26), methanol and hexane with purity ≥ 99.99%, and disposable aluminum dishes (20 mL) to be placed in the sampling chamber were all purchased from Markes International (UK). Sterile containers for feces sampling were bought from local pharmacy.

### Samples collection and preparation

A total number of 20 volunteers with different ethnicity (European, Asian, African, Latin American), all of them living in Poland at the moment of samples collection, were involved in the experiment. A written informed consent was handed to each participant, who then filled in the associated short questionnaire. The samples were collected by self-collection of feces in the sterile container provided by the researchers. The donor allotted a code to his/her own sample, which was written both on the sample container and in the questionnaire. The samples were provided in the sterile container that was wrapped in aluminum foil. All containers with samples were stored together in a freezer, while the questionnaires were placed by the donors in a blank folder. That helped in linking a sample with a given questionnaire, and in keeping anonymous the identity of donor. However, relevant information about volunteers, extracted from the questionnaires is provided in Table S1. Because some questions were not mandatory, Table S1 includes less complex information in case of some volunteers. Immediately after collecting, the samples were stored in the donor’s home fridge (between 0 and 10 h) and then transported in the lab. Here the samples were stored at − 80 °C, until the total number of 20 samples was collected (over 3 days period). Further, the samples were defrosted; from each donor 2 samples of 0.25 g were packed and prepared to be used for analysis of VOCs, while 1 g was kept for building the pool of samples. In case of 2 volunteers the sample amount was not enough to be added in the pool, while in case of one more the added amount was less than 1 g. Consequently, the pool contained the mixture of 18 samples only. The pool was very well homogenized and then divided in individual samples of 0.25 g each. The resulted samples were again stored at − 80 °C and kept until they were used for sampling of VOCs. For optimization of sampling parameters only pool samples were used, while later when VOCs screening of each donor’s sample was made. Pool samples were also analyzed in this step and used as quality controls.

### Sampling procedure using Micro-Chamber/Thermal Extractor (µ-CTE)

**Figure 1:**
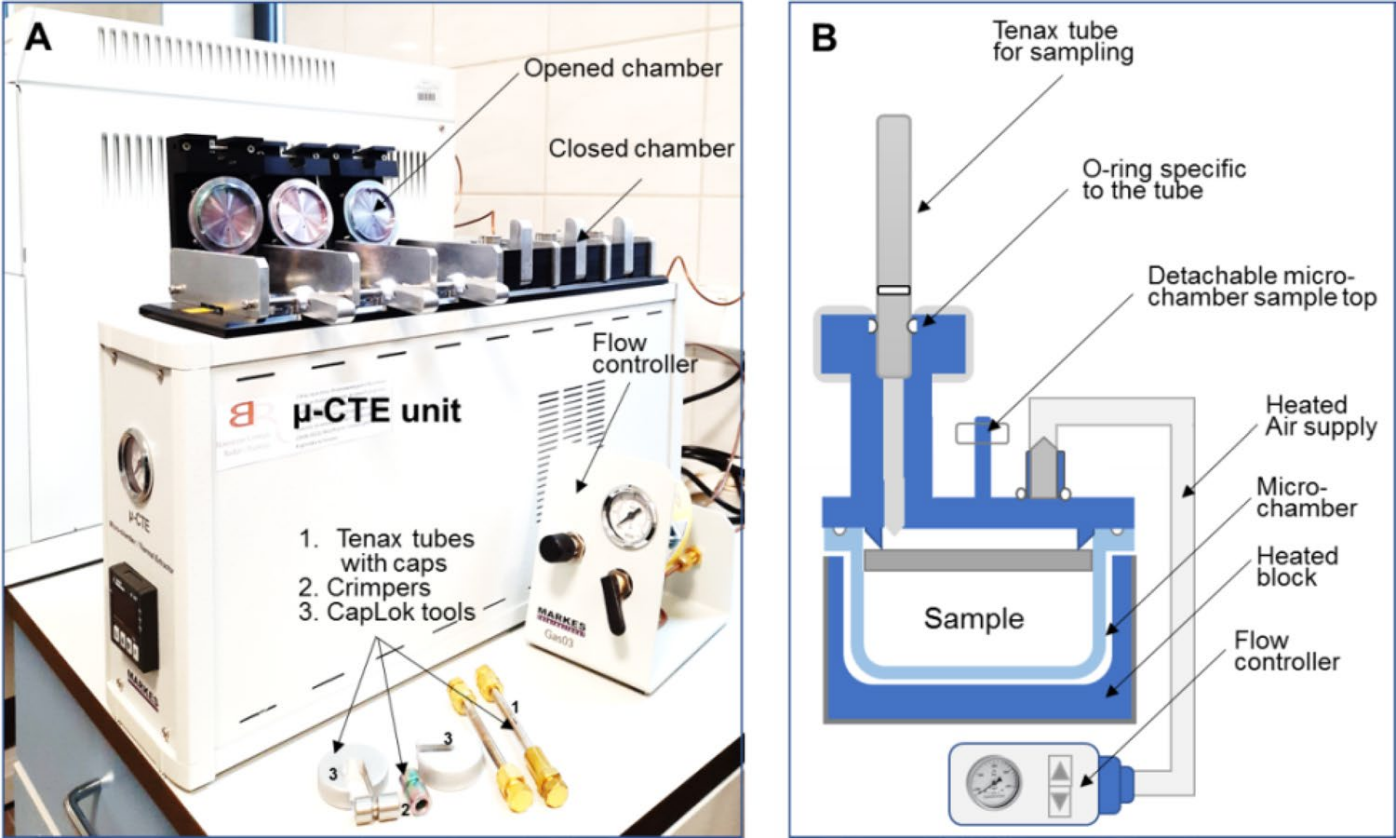
Micro-Chamber/Thermal Extractor presentation, where: (**A**) Photo presenting µ-CTE unit and accessories and (**B**) Schematic showing sampling procedure.

The Micro-Chamber/Thermal Extractor (µ-CTE), manufactured by Markes International, is a compact portable unit (31.5 cm high, 12 cm wide and 50 cm deep), with a total mass of 10.2 kg. It is equipped with up to six small cylindrical chambers (28 mm deep and 45 mm in diameter), where the sample can be placed. An adjustable flow rate (clean air, nitrogen, or helium) between 10 and 500 mL/min and temperatures from ambient up to 120 °C can be used for sampling of headspace air. The µ-CTE (Fig. 1A) was designed for sampling of semi-volatile or volatile organic compounds released from various materials, from up to six samples simultaneously.

Electrical properties are as follow: maximum power 360 W, line voltage 100–240 V, frequency 50–60 Hz, while the input inrush current is < 25 A (cold start). It can be operated at ambient temperatures between 15 to 30 °C and for relative humidity ranging from 5 to 95%^34^. Emitted components can be collected in a wide range of sorbent tubes, then analyzed by standard analytical methods (usually using a GC–MS/FID system equipped with a thermal-desorption (TD) unit).

In the present study, before sampling of headspace air, each sample of feces taken out of the freezer has been placed in the sampling chamber. Temperature and gas flow were set according with the designed runs from Design Expert software. Subsequently, the adsorbent tube (manufactured by Markes International, sorbent type: Tenax TA/Carbograph 5TD (Bio Monitoring), Stainless Steel, 1/4'' i.d., length 9 cm) was placed on the top of a sampling chamber (Fig. 1B). The gas flow made possible the passing of headspace air containing the emitted VOCs through the adsorbent tube. The Micro-Chamber/Thermal Extractor (Markes International) was connected to a pressurized gas cylinder with 99.99% nitrogen (Air Liquide, Poland). This method has achieved the dynamic head-space sampling of the VOCs associated with related metabolites. After sampling, the analyses started immediately without another further storage.

### GC–MS system used

GC–MS systems are through the most suitable analytical platforms used for VOCs analysis^24^, because some dedicated sensors, although sensitive and fast^39,40^, are providing unspecific responses^41,42^. The analysis was carried out using an Agilent 7820A GC coupled with a mass spectrometer Agilent 5977B MSD (Agilent Technologies, Waldbronn, Germany). The GC was equipped with a DB-5 MS capillary column (30 m × 0.25 mm × 1 μm). Helium was used as the carrier gas in constant flow mode, at 1 ml/min. The column temperature was programmed as follows: the initial temperature of 40 °C was held for 5 min, then the temperature was increased by 10 °C/min to 310 °C and maintained for 4 min at this value. The mass spectrometer was operating in the EI (70 eV) mode. Ion source temperature was set to 230 °C and transfer line were set to 250 °C; acquisition frequency was set at 2.9 scans/sec and mass range was 50–550 a.m.u. The dual stage thermal desorber (TD) model Markes TD-100 (Markes International, Bridgend, UK) was coupled to chromatographic column through a heated transfer line equipped with deactivated capillary (2 m × 0.25 mm) and the associated micro union. The TD conditions were as follows: sorbent tube desorption temperature 280 °C for 5 min, at desorption flow rate of 50 cm^3^ min^−1^; cold trap desorption at 300 °C for 3 min at a flow rate of 2 cm^3^ min^−1^; trap heating rate was set to 100 °C min^−1^, and transfer line temperature set to 200 °C.

### Quality control, system cleaning and stability checking

Pool samples were used as quality controls. After each four regular samples, one pool was analyzed. Every morning before starting the analyses, the instrument was subjected to a cleaning method that included graduate heating of the column up to 300 °C and purging with gas (total time of this method was 30 min). If the background level was not considered satisfactory the cleaning method was repeated. Before using them, sorbent tubes were also conditioned in the TD unit, at 320 °C for 2 h, with helium purging flow rate of 95 mL/min. Retention indexes containing a mixture of alkanes (C8 to C26, prepared in hexane) were also run daily before starting the analyses. By using the known retention times of standards, system stability was verified. Internal standard (IS) BFB (4-bromofluorobenzene) in methanol was used and added to samples at a constant concentration of 100 ng mL^−1^. After optimization of parameters, when VOCs of samples were investigated, the ratio between IS and each detected VOC was used to compare the obtained profiles. In this way, the possible errors resulting from non-reproducibility between sorbent tubes were eliminated. Eventually, the background of clean blank tubes was analyzed. The resulted components originated from blanks (coming from sorbent tubes, column bleeding and septa), pools and 20 involved volunteers, accounting for the number of 205 VOCs are shown in Table S2, presented as supplementary material. The main VOCs emitted by feces were volatile acids, hydrocarbons, alcohols, sulfur or nitrogen compounds, aldehydes and ketones.

### Statistical analyses

Response surface methodology (RSM, Design-Expert v.11, Stat-Ease, Minneapolis, Minnesota, USA), with Box-Behnken design, was used for optimization of sampling parameters as an efficient and time saving optimization method. Pearson correlation analysis and hierarchical clusters analysis were both created using IBM SPSS statistical software, package version 21. Network analysis was performed using R studio with console (version 3.6.3, Boston, MA, USA). Where applicable, Microsoft Power Point 2010 was used to label and combine the figures.

### Ethical considerations

This study was conducted according to the guidelines of the Declaration of Helsinki and approved by the Bio-Ethical Commission of Collegium Medicum in Bydgoszcz of the Nicolaus Copernicus University in Toruń, according with the agreement number KB 49/2018—16.01.2018. Written informed consent was obtained from all participants.

## Results and discussion

### Sampling optimization

#### Involved factors and fitting equations

In our study, a Box-Behnken design was utilized, in an attempt to optimize the sampling procedure for feces samples using a µ-CTE produced by Markes International. Tested parameters were as follows: extraction temperature, extraction time and gas flow rate. All of them were selected as coded independent variables (factors) at three levels: minimum (− 1), central (0) and maximum (1). The factors were tested by carrying out 17 random runs (each one in duplicate), including five central points. The used values were: temperature: 24 °C, 32 °C, 40 °C; extraction time: 10 min, 20 min and 30 min; gas flow rate: 20 mL/min, 35 mL/min and 50 mL/min. The effect of factors was observed for: number of total detected peaks, total peaks area and total intensity of the peaks. Four polynomial models: linear, two-factor interaction (2FI), quadratic and cubic model, were statistically evaluated. The linear and 2FI models were not significant, while the cubic model was aliased. This means that there were not enough unique design points to independently estimate all the coefficients for those models. Thus, the quadratic model was selected to build the response surface in the subsequent optimization process. To assess the impact of each factor on the observed responses, the second-order polynomial model [Eq. (1)] described the relation between independent variables and the obtained responses.

1 $$Y = \, \beta_{0} + \Sigma \beta_{i} X_{i} + \Sigma \beta_{ii} X_{i}^{{2}} + \Sigma \beta_{ij} X_{i} X_{j}$$

where, Y is the obtained response, X_i_ and X_j_ are the independent factors, β_0_, β_i_, β_ii_, and β_ij_ are the regression coefficients for intercept, linear, quadratic, and interaction coefficients terms, respectively.

The second order polynomial equations [Eqs. (2)–(4)] were used to express the obtained responses as a function of coded and independent variables, where Y = response; X_1_: Extraction temperature (°C); X_2_: extraction time (minutes); X_3_: flow rate (mL/min):

2 $$ \begin{aligned}{\text{Y1 }}\left[ {{\text{number}}\;{\text{ of}}\;{\text{ the }}\;{\text{ peaks}}} \right] \, & = \, { 122}.{6}0 \, {-}{ 7}.{\text{87 X}}_{{1}} + { 7}.{\text{81 X}}_{{2}} + { 4}.{\text{69 X}}_{{3}} + { 7}.{\text{37 X}}_{{1}} {\text{X}}_{{2}} \\ &\quad+ { 6}.{\text{88 X}}_{{1}} {\text{X}}_{{3}} {-}{ 7}.00{\text{ X}}_{{2}} {\text{X}}_{{3}} {-}{ 27}.0{\text{5 X}}_{{1}}^{{2}} {-}{ 5}.{\text{68 X}}_{{2}}^{{2}} + { 7}.{\text{58 X}}_{{3}}^{{2}}\end{aligned} $$

3 $$ \begin{aligned}{\text{Y2 }}\left[ {{\text{peak }}\;{\text{areas}}} \right] \, &= \, { 4}.{\text{61E}} + 0{7 }{-}{ 1}.{\text{98E}} + 0{\text{6 X}}_{{1}} + { 7}.{\text{31E}} + 0{\text{6 X}}_{{2}} + { 6}.{\text{13E}} + 0{\text{6 X}}_{{3}} {-}{ 3}.{\text{18E}} \\ &\quad+ 0{\text{6 X}}_{{1}} {\text{X}}_{{2}} {-}{ 3}.{\text{22E}} + 0{\text{6 X}}_{{1}} {\text{X}}_{{3}} {-}{ 6}.{\text{54E}} + 0{\text{6 X}}_{{2}} {\text{X}}_{{3}} {-}{ 1}.{\text{67E}} + 0{\text{7 X}}_{{1}}^{{2}} {-}{ 5}.{\text{82E}} \\ &\quad + 0{\text{6 X}}_{{2}}^{{2}} {-}{ 7}.{\text{36E}} + 0{\text{6 X}}_{{3}}^{{2}}\end{aligned} $$

4 $$ \begin{aligned}{\text{Y2 }}\left[ {{\text{peak }}\;{\text{intensity}}} \right] \, &= \, { 1}.0{\text{4E}} + 0{7 }{-}{ 4}.{\text{76E}} + 0{\text{5 X}}_{{1}} + { 1}.{\text{98E}} + 0{\text{6 X}}_{{2}} + { 1}.{\text{45E}} + 0{\text{6 X}}_{{3}} {-}{ 5}.{\text{33E}} + 0{\text{5 X}}_{{1}} {\text{X}}_{{2}} {-}{ 4}.{\text{91E}} \\ &\quad+ 0{\text{5 X}}_{{1}} {\text{X}}_{{3}} {-}{ 7}.{\text{14E}} + 0{\text{5 X}}_{{2}} {\text{X}}_{{3}} {-}{ 3}.{\text{75E}} + 0{\text{6 X}}_{{1}}^{{2}} {-}{ 3}.{\text{28E}} + 0{\text{5 X}}_{{2}}^{{2}} {-}{ 9}.{\text{42E}} + 0{\text{5 X}}_{{3}}^{{2}}\end{aligned} $$

#### Analysis of variance (ANOVA) for quadric polynomial model

For evaluation of quadratic model, criteria such as assessment of “adjusted R-squared” and “predicted R-squared” were applied. The “predicted R-Squared” was in reasonable agreement with the “adjusted R-Squared”, while the recorded difference was less than 0.2 in all three cases (see Table S3). However, the ANOVA test was used for evaluation of goodness-of-fit of the proposed design and demonstrated that all three models were significant—based on R^2^ (0.9385 to 0.9665), lack of fit (*p* > 0.05) and p-values (0.0018 to 0.0002). The R^2^ values indicate that between 93% up to 96% of the changes observed in the responses were obtained under the combination of factors and could therefore be explained by the model equations. Furthermore, low values of coefficient of variance (between 4.7 and 15.69%) were obtained, which indicates an adequate precision of the experimental values. The p-values lower than 0.0500 indicates a corresponding model, while smaller p-values are associated with more significant results. Our obtained p-values showed that there is only between 0.02% up to 0.18% chance that large F-values could occur in the experiment due to the noise. This implies that all designed quadratic models are highly significant. Lack of Fit and the obtained F values are another important criteria in the evaluation of model reliability, because regression equation and coefficient of determination are both evaluated to test the model fitting. Our obtained F values, between 0.34 and 1.05, indicate that lack of fit is not significant compared to pure error. Detailed ANOVA test results for the quadratic polynomial model are presented in Table S3.

**Figure 2:**
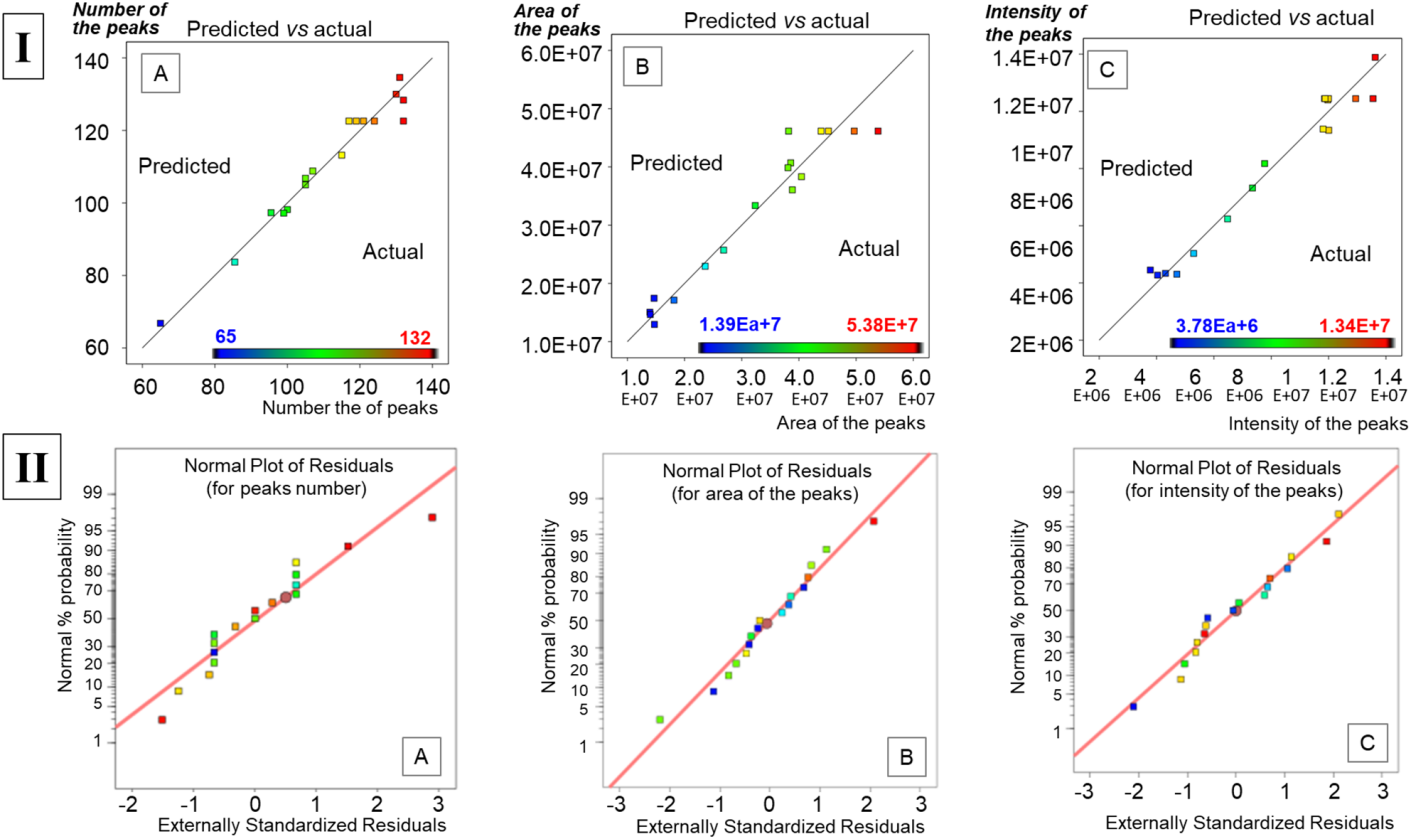
Evaluation of the adequacy of the three investigated models [created for: number of the peaks (**A**), area of the peaks (**B**) and intensity of the peaks (**C**)], where (**I**) Actual vs predicted values and (**II**) Normal plot of residuals at different production period.

The actual versus predicted responses are plotted in Fig. 2, part I. The actual values are obtained (measured) data, while the predicted values are produced by the model. By observing Fig. 2I, we can understand that all three models (A, B and C) are able to predict well the actual response. Residuals are defined as deviation between predicted and actual values. A good model is expecting that the obtained responses will follow a normal distribution along the straight line (see Fig. 2II). If an evident S-shape curve is formed, this denotes that the residuals are not following a normal distribution; consequently an inappropriate model was obtained.

According with Fig. 2II, the Studentized residuals follow a normal distribution. The results presented in Table S3 and Fig. 2 indicate that there is not any evidence pointing out possible errors regarding the adequacy of the model.

#### The influence of investigated factors on the obtained responses

##### Single factor influence

**Figure 3:**
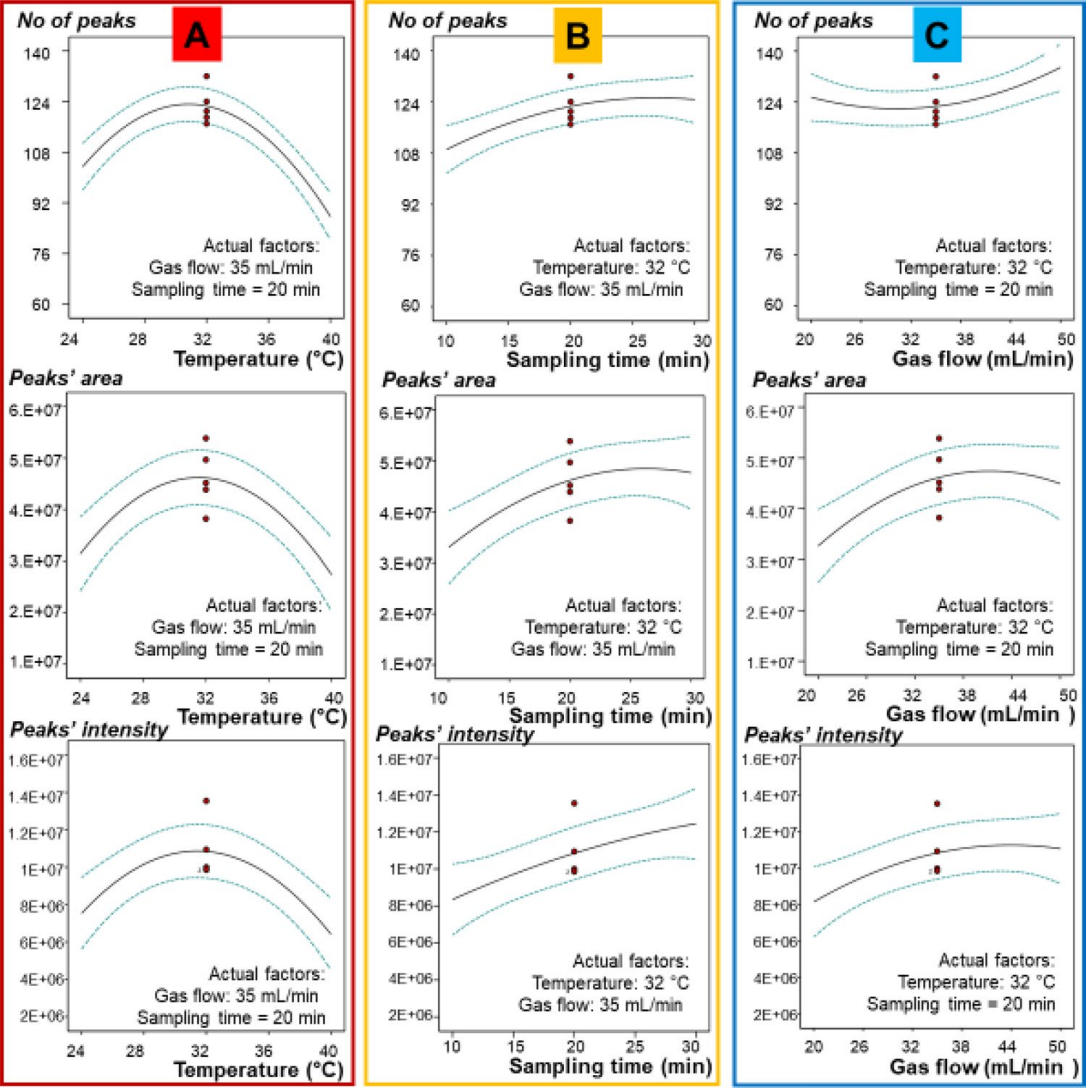
Influence of investigated factors on the obtained responses, where: (**A**) influence of temperature, (**B**) influence of extraction time and (**C**) influence of gas flow).

The influence of each investigated factor on the obtained responses is depicted in Fig. 3. Part A is presenting the influence of temperature variation when extraction time and gas flow rate were kept constant at central points (extraction time 20 min and gas flow rate 35 mL/min). As highlighted, for all three models the best responses were obtained around the value of 32 °C. Presumably, this means that lowest temperature (24 °C) in not enough to fully volatilize the components, while the highest (i.e. 40 °C) can produce a series of problems that lead either to the loss of volatiles, to their conversion in different types of compounds and/or to some other specific issues. For example, it was reported that microbial fermentation processes are accelerated at higher temperatures^43,44^, while different patterns of amino-acids decomposition by bacteria in biological samples were also referred^16,19^. However, since VOCs have specific optimal temperatures of volatilization, the choice of optimal temperature depends on the VOC of interest, which may differ according to the desired application and to the system used for analysis. In our case, we could observe in the chromatograms of samples analyzed at 40 °C the presence of unseparated (overlapped) wide peaks. Nevertheless, in the mentioned chromatograms both peaks intensity and peaks area were lower, when compared with the chromatograms recorded at lower temperatures. Regarding the targeted VOCs, we were more interested in highly volatile short chain alcohols, acids and other compounds containing nitrogen and sulfur.

In Fig. 3B the influence of extraction time is presented when temperature and gas flow rate were constant (temperature 32 °C and gas flow rate 35 mL/min). It can be observed that in all three cases extraction time has a direct influence on the obtained responses, with longer extraction time giving higher responses. In case of number of the peaks and peaks area, the trend line arrived at the plateau, while in case of peaks intensity it is still increasing. This presumably can mean that a longer extraction time could have given a higher response. When gas flow rate was investigated at constant extraction time (20 min) and temperature (32 °C), the trend line was rising with increasing flow rate in case of peaks number (Fig. 3C). In case of peaks area, the plateau was reached between 38 and 44 mL/min, followed by a slight decrease of the trend line when the flow rate was increased above 45 mL/min. However, a tendency of continuous increasing was observed in case of peaks intensity, with plateau tentative at the maximum flow rate. Consequently, we can state in this step that longer extraction time as well as higher gas flow rate and temperature around 30 °C will facilitate obtaining of the best possible results.

##### Two factors influence

**Figure 4:**
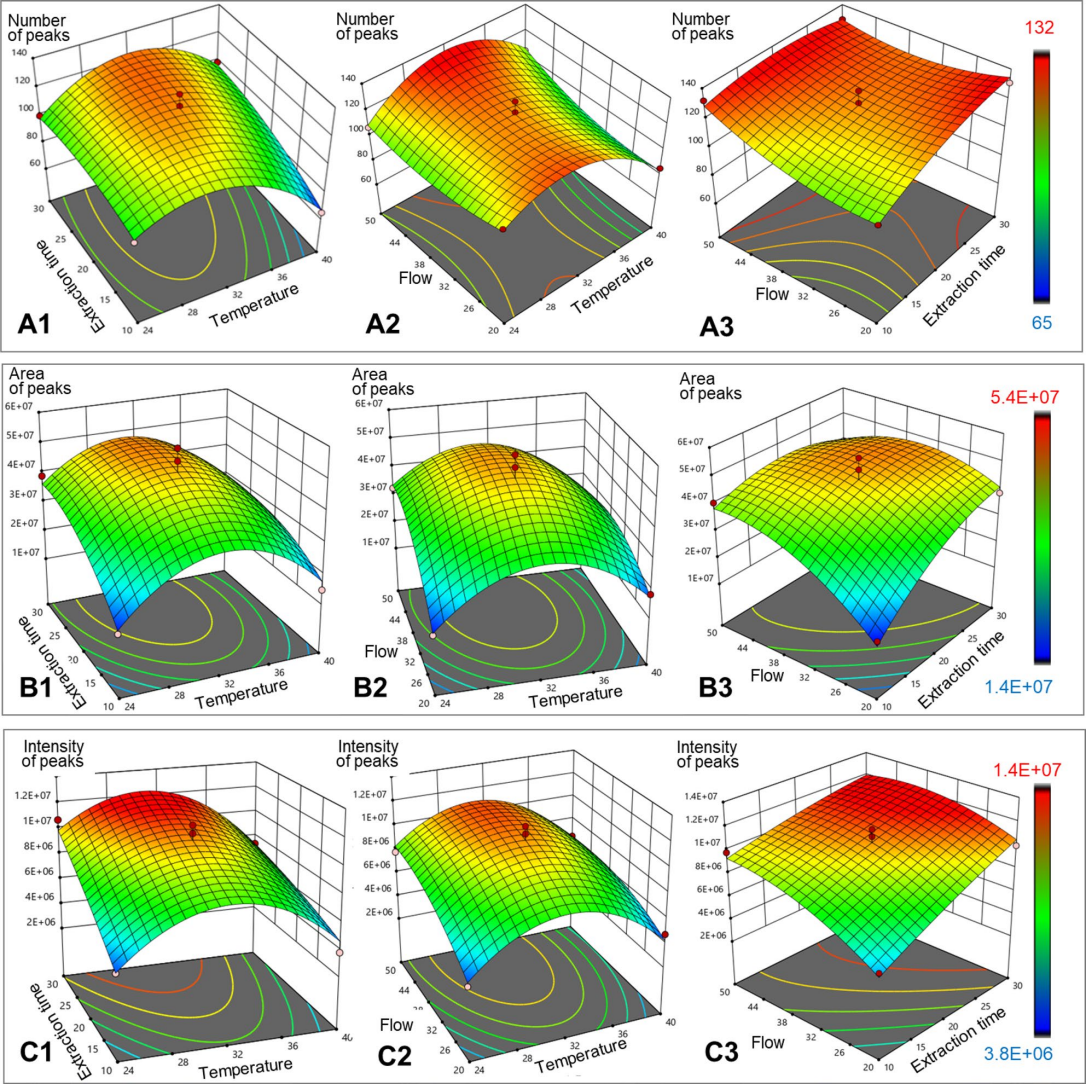
3D surface response for interaction effect of two-parameters according with: (**A**) number of the peaks (**A1**—extraction time vs temperature, **A2**—flow vs temperature, **A3**—flow vs extraction time), (**B**) area of the peaks (**B1**—extraction time vs temperature, **B2**—flow vs temperature, **B3**—flow vs extraction time), (**C**) intensity of the peaks (**C1**—extraction time vs temperature, **C2**—flow vs temperature, **C3**—flow vs extraction time).

Preliminary solutions were obtained after single factor influence checking. Nevertheless, the main goal of our study was to develop a method that can provide the best solution to be used as a combination of the mentioned factors, once in the sampling process the variables can be influenced between themselves. Consequently, two factors influence, when a third one (named actual factor) is kept constant at the central point, is highlighted in Fig. 4 and will be further discussed. In case of peaks number (Fig. 4A), when the actual factor was gas flow rate (kept constant at 35 mL/min), it was observed that a middle temperature (around 30 °C) and middle extraction time (approximatively 20 min) provided the higher response (A1). By maintaining the extraction time constant then middle temperature and high gas flow rate (> 45 mL/min) provided the best solutions (A2). Eventually, at constant temperature (32 °C) it was observed that highest gas flow (tending to the maximum value) could lead to finding the maximum possible number of peaks, while the extraction time did not present significant influence (A3). These findings indicate that in case of number of peaks found, the most relevant factor is flow rate. Moreover, by observing the last “umbrella” of Fig. 4A (A3), one may conclude that the usage of higher gas flow could have led to a higher number of found peaks.

For peaks area (Fig. 4B), a very good fitted model was obtained, once the figures shape is like well-contoured umbrellas, and the color scale in going from the lowest to the highest values (blue to red, respectively). It was highlighted that when the actual factor was gas flow rate, an extraction time between 25 and 30 min at about 32 °C is giving a good response (B1). If the actual factor was considered extraction time, a temperature around 30 °C and a gas flow rate of 40 mL/min provided the highest response (B2). In the third case (B3), when the actual factor was temperature, the highest extraction time (30 min), when using a gas flow rate between 35 and 40 mL/min provided the highest peaks area. By observing Fig. 4B3 it is highlighted that longer extraction times could have led to the recording of higher peak areas. In case of this model, gas flow rate and extraction time were more influential factors than temperature.

Finally, the influence of factors on peaks intensity is presented in Fig. 4C. Part C1 (gas flow rate = constant at 35 mL/min) is showing that at highest extraction time (30 min) and middle temperature (around 30 °C) is leading to the highest intensities. Moreover, it may be observed that in Part C1 that longer extraction time can provide peaks of higher intensity. Nevertheless, high gas flow rate (50 mL/min) and temperature near to 30 °C provided good solution, although a gas flow rate > 50 mL/min would probably have produced more intense peaks, as shown in Part C2. It can be observed in Part C3 (where the actual factor was temperature) that a gas flow between 35 and 45 mL/min and a extraction time ≥ 30 min led to obtaining of maximum intensity, although a higher extraction time could have resulted in more intense peaks. These findings suggest that in case of peaks intensity the most relevant factor was extraction time.

#### Obtained solutions

The final obtained solutions were predicted by the software as a result of experimental measurements and total interactions between the three selected factors. For optimization the experimental parameters and obtaining the results, the steps were as follows:

- initial values (minimum, medium, maximum) were established for all three factors;
- the values were introduced, and then the software generated—based on the given values—a combination of 17 experimental conditions;
- the 17 experiments were run and a response was obtained for each experiment;
- going back to the software, the 17 obtained responses were introduced; based on them, the Design Expert Software calculated the predicted responses;
- using the predicted solutions, samples were run to verify the validity of the prediction.

The predicted solutions, that were verified experimentally for each model, are presented in Table 1.

**Table 1:** Obtained solutions as a result of optimization of sampling parameters.

Approach	Temp (°C)	Extrac-tion time (min)	Gas flow (mL/min)	Predicted response	Standard error	Desirability	Measured response		
							R1 (no of peaks)	R2 (area of peaks)	R3 (intensity of peaks)
Number of peaks	**30.661**	**19.612**	**48.687**	133	3	1.000	**137**	**4.60E+07**	**1.31E+07**
Area of peaks	30.948	25.528	38.004	4.89E+07	2.25E+06	0.936	128	5.51E+07	1.67E+07
Intensity of peaks	30.572	29.242	46.887	1.23E+07	5.96E+05	1.000	91	4.41E+07	1.17E+07

R1—the obtained response when the solution acquired for peaks number was used.

R2—the obtained response when the solution acquired for peaks area was used.

R3—the obtained response when the solution acquired for peaks intensity was used.

Because the software affords for selection of solutions (in term of goal and importance), thus allowing the prioritization of options according to needs, certain criteria were chosen in attempt to obtain the best solutions that will fit our model. Thereby, maximum importance was accorded to each factor (temperature, extraction time and gas flow), while the selected goal was “in range”. We assigned also maximum importance to the obtained response and the selected goal was “maximize”, while in case of error the selected goal was “in range” and the selected importance was medium. According to Table 1, the most suitable parameters to be used are the parameters obtained according with peak numbers (highlighted in bold).

### Testing of developed model

**Figure 5:**
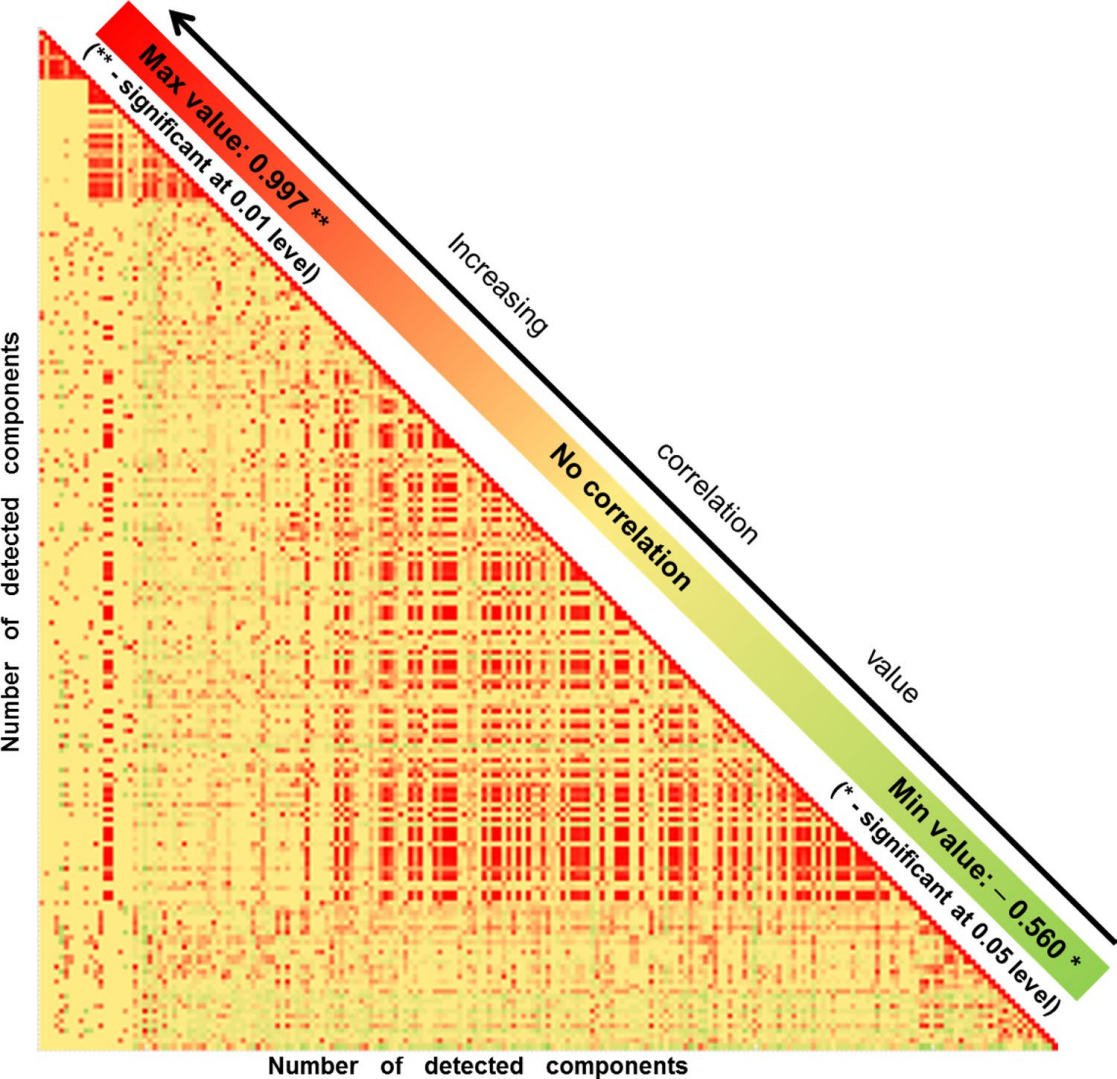
Correlation matrix presenting the significance of the 205 detected VOCs, highlighted in the form of a heat map whose shades represent the correlation values (green—negative correlation, yellow—no correlation, red—positive correlation).

**Figure 6:**
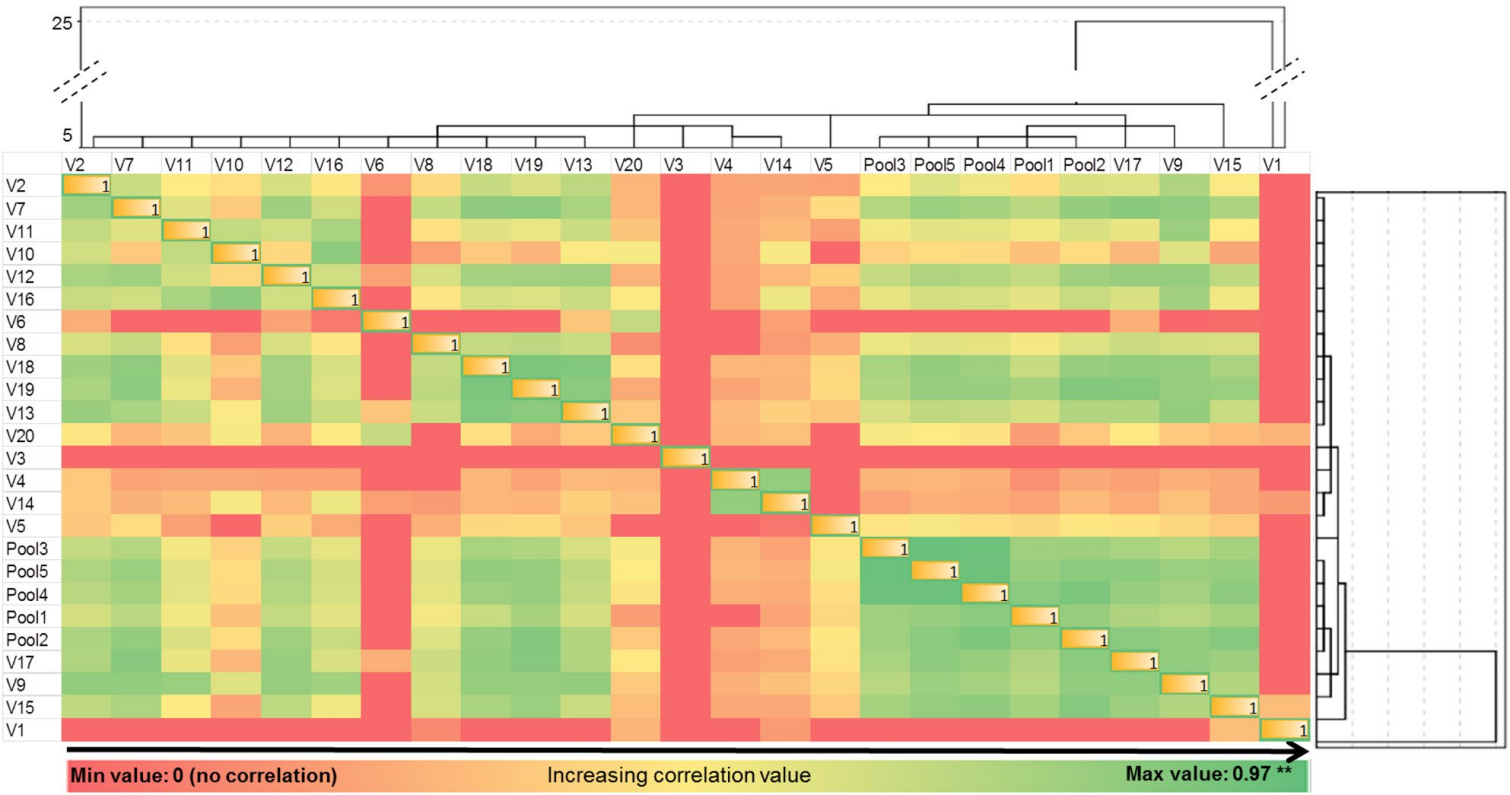
Heat map representing the correlation between the investigated variables according with hierarchical clusters analyses.

**Figure 7:**
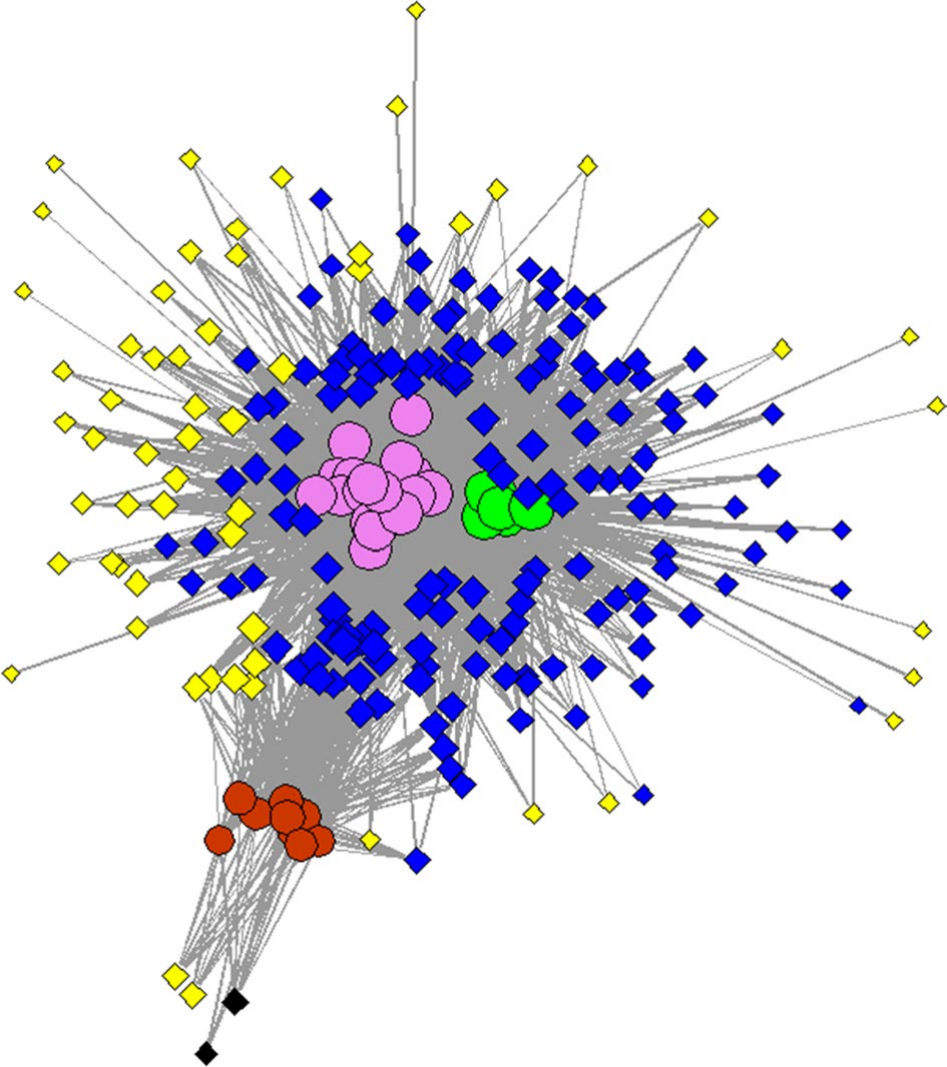
Network analyses representing separation between the three investigated groups based on the recorder VOCs profiles coming from pool samples (green circles), included volunteers (purple circles) and blank tubes (red circles). The blue diamonds are representing the components common between pools and volunteers, the yellow diamonds are highlighting the VOCs appearing in volunteers only, while the black ones were detected in blanks only.

In order to test the validity of the developed model, Correlation Analysis and Network Analysis were run. In case of correlation, parametric tests were used once they are “a statistical tool expecting a linear correlation between the investigated variables coming from the same source, and/or they have the same measure unit”^45^. Two different approaches were tackled: correlation between all 205 detected components in the whole matrix (including detected components appearing in pool samples, in samples from each volunteer and in blanks)—represented in Fig. 5; and correlation between all 20 included volunteers and pool samples—as presented in Fig. 6. Network analysis highlighted in Fig. 7 is presenting discrimination between the investigated groups, act that confirms the validity of the model.

### Correlation based on detected components

A Pearson moment product correlation was used to highlight the correlation that occurs between the detected components. Because the matrix was complex (including 205 VOCs), only a snap shot representation could be presented in Fig. 5, in the form of a heat map. The correlation values significant at 0.01 level were between r(205) =  – 446 to 0.999, which pointed out in a low up to very strong positive or negative correlation. Moreover, the correlation values significant at a 0.05 level accounted for r(205) = – 0.560 to 0.426, denotative of a moderate correlation.

The strong and very strong correlation that was achieved when p was significant at 0.01 level (p = 0.01), was a positive correlation. All the components detected in blanks presented a positive correlation with each other (r(205) = 0.145, p = 0.05 up to 0.982, p = 0.01). With few exceptions, discussed below, the components detected in pooled samples (accounting the number of 146) positively correlated with all the detected components. Examples of compounds that did not present any correlation with the pool samples, but they were however highlighted as components originated from blank tubes, were ethyl acetate and 1,3,5-trifluorobenzene. Some other examples were those VOCs detected in case of limited number of volunteers only, and in relatively small amounts, reasons why they were not detectable in pool samples.

Less often than positive correlation, a negative correlation (r(205) = – 0.56, up to – 0.15, p = 0.05) was also observed. Components that exhibited the most often a negative correlation with other present in the matrix were: 2-propanamine 2-methyl-; fumaronitrile; trifluoromethyltrimethylsilane; 2,5-hexanediol; cyclotetrasiloxane, octamethyl-; cyclopentasiloxane, decamethyl-; biphenyl; dibutyl phthalate; cyclohexane, isothiocyanato-; heptasiloxane, 1,1,3,3,5,5,7,7,9,9,11,11,13,13-benzene, 1,1′-(4,4-dimethyl-1-butene-1,4-diyl)bis-tetradecamethyl- and five other compounds that could not be identified and remained unknown. They were generally associated with components coming from tubes, septa and columns bleeding. These findings suggest that the background may affect the obtained results; however, generally low values of negative correlation were observed.

However, the most of the VOCs common between pooled samples and volunteers’ samples presented moderate or low positive correlation. According with these findings we can assume the validity of the model, while we find positive correlation between samples and pools when investigating the detected output (the VOCs). This denotes also that the model worked correctly and that the pools were representative.

#### Correlation based on investigated samples

As a second approach, a Pearson moment correlation was conducted also in order to check the correlation and the level of significance between pool samples and samples coming from each volunteer. The whole VOCs profile of each sample was used in this approach. The correlation matrix was designed based on the hierarchical clusters’ analyses, obtained using the method “average linkage between groups” within the interval “squared Euclidian distance” and presented in Fig. 6.

The dendrogram of clusters’ analysis shows the formation of seven main clusters with different levels of significance. The samples coming from 11 volunteers fused together in one cluster with similar distance level in the left part of dendrogram, a finding suggesting that they had a similar VOCs composition. The samples #20, #3, #4, #14 and #5 clustered next to them, highlighting less similarities compared with the first 11 already mentioned. Nevertheless, positioned at the opposite site, in a rather arbitrarily manner, were the clusters corresponding to volunteers #17,#9, #15 and #1, all of them with different levels of significance. These denote that the VOCs composition of the mentioned samples have presented the most different features when compared with others. All five pool samples with the same level of similarity were segregated, between the other clusters, drawing actually the border between those with similar VOCs profiles and those that presented most different features. Observing pools positioning in Fig. 6, we can assume that they were representative for the batch of analyzed samples, and consequently we presume also the validity of the developed model, when the analyzed samples were considered as variables.

Regarding the heat map, it can be observed that the highest correlation occurred, as expected, between the pool samples (r(23) = 0.762 up to 0.970, p = 0.01). The pools were also well correlated with the samples. However, they were less correlated with the samples coming from volunteers #1, #3 and #6. These samples were associated with the three cases where the amount of sample was insufficient to be added to the pool. Except of this, positive correlation from low to very high was accounted between samples originated from volunteers. As a general conclusion, all these findings indicate that there is a relevant correlation between the investigated samples, both when the investigated variables were the detected VOCs and when each sample was investigated based on the entire profile, even if these characteristics are far different and in appearance not linked-up with each other.

#### Discrimination between the groups based on network analysis

The dataset representing distributions of the 205 detected VOCs was used to build the network analysis model (Fig. 7). Consequently, based on the obtained profiles and using the incidence of the peaks, a network analysis was created in order to obtain an exploration of the data. R studio with console (version 3.6.3, Boston, MA, USA) was used for network analyses. The model separated the three investigated groups, by leading into the formation of three cluster groups: the group of pools, the group of volunteers and the blanks.

In addition, VOCs detected just in volunteers’ samples have been dispersed around the mentioned group (purple circles), common VOCs between pools and volunteers were located mostly between the two groups, and the VOCs detected in blanks were scattered into the left-down part (black diamonds). Regarding the number of VOCs used in network analysis, 2 were specific for blanks, 59 for volunteers, while 146 VOCs were common between the pools and volunteers. We presume that most of VOCs presented in Fig. 7 are endogenous, being therefore generated by the organism as a normal process of its metabolism. However, certainly part of VOCs are exogenous, being absorbed by the organism from the environment and then eliminated through feces. However, variability in VOCs coming from different subjects was observed, and this phenomenon is related to several factors, among which we could mention diet, living style, personal habits, etc.

### The possible origins of VOCs detected in the investigated feces samples

The 205 VOCs detected in feces samples may have different origins. Most of them are human metabolic products or may have gut microbiota origins, while others can be exogenous components and contaminants coming from the analytical platform used. However, as a general rule VOC profiles emitted by feces are directly connected with processes induced by gut microbiota, processes that can change in case of diseases. For example, it was proven that the analysis of fecal samples coming from 30 healthy volunteers provided 80% of similar VOCs, but significant changes were found in those samples collected from patients with *Clostridium difficile, Campylobacter jejuni* and ulcerative colitis^26,46^. Also, the VOCs emitted from feces proved significant evidence of disease etiology, as well as the association of some biomarkers with specific diseases^47,48^. For instance, infectious with *Clostridium difficile* resulted in furans identification; the rotaviruses generated ethyl dodecanoate, while enteric viruses produced ammonia^47^.

We assume that the primary source of VOCs in feces are the carbohydrates that are metabolized by gut microbiota into pyruvate, which is further transformed into ethanol or acetic acid and other by-products (1-propanol, 2-propanol or 1-butanol) through the fermentation processes^49^. Moreover, amino-acids decomposition could be the source of VOCs like 3-methyl-1-butanol, 3-methylbutanal, or 3-methylbutanoic acid^47^. Moreover, certainly gut microbiota have converted tryptophan to indole^50^ and cysteine or methionine to sulphur VOCs (dimethyl disulfide, dimethyl trisulfide or dimethyl tetrasulfide); all these VOCs were detected in our study. Triglycerides hydrolysis is resulting in formation of fatty acids, and then fatty acids decomposition by gut microbiota can in turn lead to formation of various hydrocarbons, aliphatics, ketones, and other VOCs^47^. Nevertheless, methyl ketones are the end products of fatty acids decarboxylation process^50^.

## Conclusions

An optimized sampling method for VOCs emitted from feces by using as sampling system a Micro-Chamber/Thermal Extractor (µ-CTE), method able to provide the samples collection in controlled conditions was developed in the present study. Design Expert software (with Box–Behnken design) was used as simulation software for predicting the solutions that were eventually verified experimentally. The developed model proved to be correct according with statistical analysis (ANOVA tests, Actual vs Predicted Values and Normal plot of Residuals). Consequently, the extraction time of 19.6 min, at a extraction temperature of 30.6 °C by using a flow rate of 48.7 mL/min provided the higher response. The developed method was validated by using correlation tests and network analysis, that both proved the validity of the developed model. By investigating correlation based on detected components (Fig. 5) we concluded that the components detected in pools positively correlated with all the detected components in the samples. This proves that as higher are those components in samples they will positively impact the pools. When searching into correlation based on investigated samples (Fig. 6) we concluded that there was variability between investigated samples. However, by obtaining all pools segregated in the same cluster, we proved that they were representative for the given batch. Finally, network analysis (Fig. 7) successfully separated the group of pools, group of volunteers and the blanks, fact that confirms that the developed optimization was applied to a well-chosen and representative group of samples.

## Supplementary Information

Supplementary Table S1.

Supplementary Table S2.

Supplementary Table S3.

## Abbreviation

μ-CTE: Micro-Chamber/Thermal Extractor
