# Supplementary material for: Micro-Chamber/Thermal Extractor (µ-CTE) as a new sampling system for VOCs emitted by feces
Source: Sci Rep. 2021 Sep 21;11:18780. doi: 10.1038/s41598-021-98279-z (PMC8455535; doi:10.1038/s41598-021-98279-z)
Supplement: Supplementary file 1 — Supplementary Table S1. [file 41598_2021_98279_MOESM1_ESM.docx]

Table S1. Data related to included volunteers

| **Volunteers ID**  **(details)** | **With self-declared chronical diseases/ Administrated medication in the last month *** | **Alcohol~ / coffee #** | **Liquids ^** **/frequency of stools per day** | **Type of diet in preferred (aliments consumed 24 hours before sample collection)** |
| --- | --- | --- | --- | --- |
| V1: female, 35 years old, passive smoker | Not/ probiotics and / or dietary supplements, other | Yes/ yes (2 cups) | 1 L / 1 | Balanced including proteins, carb and fat (vegetables, bread, meat, milk, fruits) |
| V2: female (49 kg, 1.61 cm), 28 years old, occasionally smoker | Not/ other (contraceptives) | Not/ yes (3 cups) | 1-1.5 L / 1 | Balanced including proteins, carb and fat (chicken meat, rice, salad, yogurt, banana) |
| V3: female (64 kg, 1.70 cm), 64 years old, non-smoker | Not/ other | Yes/ yes (2 cups) | 3 L / 1 | Balanced including proteins, carb and fat (sausages, vegetable soup, meat, bread, potatoes, butter) |
| V4: male (80 kg, 1.80 cm), 67 years old, smoker (3 cigarettes /day) | Not/ other | Yes/ yes (2 cups) | 2 L / 1 | Balanced including proteins, carb and fat (vegetable soup, meat with vegetables, bread, potatoes, butter) |
| V5: female (95 kg, 1.75 cm), 32 years old, passive smoker | Yes/ probiotics and / or dietary supplements, anti-inflammatory, other | Not/ yes (0.5 cup) | 1.5 l / 1 to 2 | Balanced including proteins, carb and fat (rice, smoked fish, bread, soft cheese, vegetables (onion, green salad, cucumber, tomato, cabbage, avocado, algae) oil, chocolate cookies, milk, vegetable soup with meat and beans). |
| V6: female (80 kg, 1.69 cm), 36 years old, smoker (10 to 12 cigarettes /day) | Not/ not applicable | Not/ yes (1 to 2 cups) | 1 L / 1 to 2 | Balanced including proteins, carb and fat (yogurt with corn flakes, vegetable soup, rice with meat, vegetable salad (cabbage, tomato, red peppers, cucumber), fruits (apples, blueberries, grape, almonds), milk). |
| V7: female (58 kg, 1.66 cm), 39 years old, smoker (5 cigarettes / day) | Not/ other | Yes/ yes  (1 cup) | 2 L / 1 | Balanced including proteins, carb and fat (chicken meat, bread, curry, milk) |
| V8: male (72 kg, 1.70 cm), 48 years old, non-smoker | Not/ probiotics | Yes/ yes (0.5 cup) | 2 L / 1 | Balanced including proteins, carb and fat (cheese, bread, butter, meat, pasta) |
| V9: female (92 kg, 1.69 cm), 51 years old, non-smoker | Yes/ other (related to the thyroid gland) | No/ yes (2 cups) | 1.5 / 1 | Balanced including proteins, carb and fat (eggs, bread, cucumber, fish, potatoes, salad, cheese, butter, wheat roll, tomato, apple, banana, tea) |
| V10: male (120 kg, 1.94 cm), 21 years old, non-smoker | Not/ not applicable | Not/ (1 to 2 cups) | 3 - 4 L/  2 to 3 | High carb, low fat (fish, sausages, beans, bread, mini pies with ham, peppers, cheese, cake (royal pie), milk (cafe latte). |
| V11: male (102 kg, 1.76 cm), 51 years old, non-smoker | Not/ not applicable | Not/ not | 1.5 L/ 1 | Balanced including proteins, carb and fat (scrambled eggs, bread, fish, potatoes (boiled), salad) |
| V12: male (100 kg, 1.81 cm), 69 years old, former smoker | Yes/ probiotics and / or dietary supplements, other | Not/ yes (2 cups) | 1 L / 1 to 2 | Both high carb, low fat & Balanced including proteins, carb and fat (bread, cheese, meat, ham, vegetables, fruits) |
| V13: female (80 kg, 1.56 cm), 64 years old, non-smoker | Not/ probiotics and / or dietary supplements | Not/ yes (2 cups) | 1 L / 1 to 2 | Balanced including proteins, carb and fat (cheese, bread, fried meat, ham, vegetables, fruits, potatoes, juice) |
| V14: female (56 kg, 1.64 cm), 31 years old, non-smoker | Not/ other (contraceptives) | Yes/ (1 to 3 cups) | 2 L/ 1 to 2 | Balanced including proteins, carb and fat (rye bread, ham, yogurt, pasta with tomato sauce (including beef meat, onion and red pepper), strawberries, milk) |
| V15: male (71 kg, 1.67 cm), 66 years old, non-smoker | Not/ not applicable | Yes/ (1 cups) | 2 L/ 1 | Balanced including proteins, carb and fat |
| V16: male (57 kg, 1.64 cm), 22 years old, non-smoker | Not/ not applicable | Not/ yes (1 cup) | 2 L/ 1 | High protein (rice, soy, chicken, cabbage, mushrooms, green pepper, onion, garlic, carrot, tomato, milk, apple) |
| V17: male (75 kg, 1.74 cm), 33 years old, non-smoker | Not/ not applicable | Not/ yes (3 cup) | 2 L/ 2 per week | Other – high carb, high fat (bread, chocolate cream with nuts, fish, potatoes, chocolate, tea) |
| V 18: male (73 kg, 1.68 cm), 69 years old, non-smoker | Not/ not applicable | Yes/ yes (1 cup) | 2 L / 1 | Balanced including proteins, carb and fat (bread, meat, potatoes, vegetables, fruits) |
| V19: female (60 kg, 1.62 cm), 47 years old, non-smoker | Not/ not applicable | Not / yes (0.5 cup) | 2 L / 1 | Low carb, high fat (bread, butter, meat, rice) |
| V20: female (90 kg, 1.63 cm), 31 years old, non-smoker | Not/ anti-inflammatory | Not/ yes (2 to 3 cups) | 1.5 L/ 1 every two days | High carb, low fat (cereals with yogurt, banana, fried egg, cheese, tomato, radish, toast bread, strawberries, sandwiches (including salmon, garlic & lemon juice)) |

*** -** the options were: antibiotics, anti-inflammatory, probiotics and/or dietary supplements, other (possibly what).

~ - Alcohol consumption three days before samples collection (just yes or not)

# **-** how many cups are consumed usually per day; 1 cup was considered as maximum 200 mL.

^ **-** Liquids consumption 24 h before samples collection
