# Supplementary material for: Micro-Chamber/Thermal Extractor (µ-CTE) as a new sampling system for VOCs emitted by feces
Source: Sci Rep. 2021 Sep 21;11:18780. doi: 10.1038/s41598-021-98279-z (PMC8455535; doi:10.1038/s41598-021-98279-z)
Supplement: Supplementary file 3 — Supplementary Table S3. [file 41598_2021_98279_MOESM3_ESM.docx]

Table S3 Analyses of variance for the fitted quadratic polynomial model

| **Source** | **Sum of squares** | **DF** | **Mean square** | **F-value** | **p-value** | **Observation** |
| --- | --- | --- | --- | --- | --- | --- |
| **Optimization results function of peak numbers** | | | | | | |
| Model | 5196.80 | 9 | 577.42 | 21.30 | 0.0003 | significant |
| Residual | 189.76 | 7 | 27.11 |  |  |  |
| Lack of Fit | 52.56 | 3 | 17.52 | 0.5108 | 0.6961 | not significant |
| Pure Error | 137.20 | 4 | 34.30 |  |  |  |
| Cor Total | 5386.56 | 16 |  |  |  |  |
| Std. Dev. | 5.21 |  |  |  |  |  |
| Mean | 110.76 |  |  |  |  |  |
| C.V. % | 4.70 |  |  |  |  |  |
| R² | 0.9648 |  |  |  |  |  |
| Adjusted R² | 0.9195 |  |  |  |  |  |
| Predicted R² | 0.8041 |  |  |  |  |  |
| Adeq. Precision | 16.9816 |  |  |  |  |  |
| **Optimization results function of peak areas** | | | | | | |
| Model | 2.701E+15 | 9 | 3.002E+14 | 11.86 | 0.0018 | significant |
| Residual | 1.771E+14 | 7 | 2.531E+13 |  |  |  |
| Lack of Fit | 3.620E+13 | 3 | 1.207E+13 | 0.3425 | 0.7973 | not significant |
| Pure Error | 1.409E+14 | 4 | 3.523E+13 |  |  |  |
| Cor Total | 2.878E+15 | 16 |  |  |  |  |
| Std. Dev. | 5.030E+06 |  |  |  |  |  |
| Mean | 3.206E+07 |  |  |  |  |  |
| C.V. % | 15.69 |  |  |  |  |  |
| R² | 0.9385 |  |  |  |  |  |
| Adjusted R² | 0.8593 |  |  |  |  |  |
| Predicted R² | 0.7223 |  |  |  |  |  |
| Adeq. Precision | 8.6025 |  |  |  |  |  |
| **Optimization results function of peak intensities** | | | | | | |
| Model | 1.203E+14 | 9 | 1.337E+13 | 22.45 | 0.0002 | significant |
| **Residual** | 4.167E+12 | 7 | 5.953E+11 |  |  |  |
| Lack of Fit | 1.831E+12 | 3 | 6.105E+11 | 1.05 | 0.4637 | not significant |
| Pure Error | 2.336E+12 | 4 | 5.840E+11 |  |  |  |
| **Cor Total** | 1.245E+14 | 16 |  |  |  |  |
| Std. Dev. | 7.716E+05 |  |  |  |  |  |
| Mean | 8.081E+06 |  |  |  |  |  |
| C.V. % | 9.55 |  |  |  |  |  |
| R² | 0.9665 |  |  |  |  |  |
| Adjusted R² | 0.9235 |  |  |  |  |  |
| Predicted R² | 0.7352 |  |  |  |  |  |
| Adeq Precision | 12.8633 |  |  |  |  |  |

DF – degrees of freedom; C.V.%: Coefficient of variance (%).
